# Boysenberry polyphenol inhibits endothelial dysfunction and improves vascular health

**DOI:** 10.1371/journal.pone.0202051

**Published:** 2018-08-14

**Authors:** Ryo Furuuchi, Ippei Shimizu, Yohko Yoshida, Yuka Hayashi, Ryutaro Ikegami, Masayoshi Suda, Goro Katsuumi, Takayuki Wakasugi, Masaaki Nakao, Tohru Minamino

**Affiliations:** 1 Department of Cardiovascular Biology and Medicine, Niigata University Graduate School of Medical and Dental Sciences, Niigata, Japan; 2 Bourbon Corporation, Niigata, Japan; 3 Division of Molecular Aging and Cell Biology, Niigata University Graduate School of Medical and Dental Sciences, Niigata, Japan; Osaka University Graduate School of Medicine, JAPAN

## Abstract

Endothelial cells have an important role in maintaining vascular homeostasis. Age-related disorders (including obesity, diabetes, and hypertension) or aging *per se* induce endothelial dysfunction that predisposes to the development of atherosclerosis. Polyphenols have been reported to suppress age-related endothelial cell disorders, but their role in vascular function is yet to be determined. We investigated the influence of boysenberry polyphenol on vascular health under metabolic stress in a murine model of dietary obesity. We found that administration of boysenberry polyphenol suppressed production of reactive oxygen species (ROS) and increased production of nitric oxide (NO) in the aorta. It has been reported that p53 induces cellular senescence and has a crucial role in age-related disorders, including heart failure and diabetes. Administration of boysenberry polyphenol significantly reduced the endothelial p53 level in the aorta and ameliorated endothelial cell dysfunction in iliac arteries under metabolic stress. Boysenberry polyphenol also reduced ROS and p53 levels in cultured human umbilical vein endothelial cells (HUVECs), while increasing NO production. Uncoupled endothelial nitric oxide synthase (eNOS monomer) is known to promote ROS production. We found that boysenberry polyphenol reduced eNOS monomer levels both in vivo and in vitro, along with an increase of eNOS dimerization. To investigate the components of boysenberry polyphenol mediating these favorable biological effects, we extracted the anthocyanin fractions. We found that anthocyanins contributed to suppression of ROS and p53, in association with increased NO production and eNOS dimerization. In an ex vivo study, anthocyanins promoted relaxation of iliac arteries from mice with dietary obesity. These findings indicate that boysenberry polyphenol and anthocyanins, a major component of this polyphenol, inhibit endothelial dysfunction and contribute to maintenance of vascular homeostasis.

## Introduction

Aging leads to an increasing prevalence of age-related disorders, such as diabetes and heart failure. Aging is a complex phenomenon and is difficult to fully comprehend, but studies have suggested that cellular senescence is pivotal to the progression of various undesirable aspects of aging. Decades ago, fibroblasts were shown to have limited replication potential [1], suggesting that aging also occurs at the cellular level, which is described as “cellular senescence”. Senescent cells are characterized by enlargement, growth arrest, and alterations of gene expression. The changes of gene expression make these cells resistant to apoptosis and lead to secretion of pro-inflammatory molecules, contributing to the development of chronic inflammation and tissue remodeling [2].

Aging and lifestyle-related diseases induce vascular dysfunction, and increases the risk of cardiovascular disease [3, 4]. Cellular senescence also affects the vasculature. This is termed “vascular senescence” and is well recognized to promote cardiovascular disorders, including atherosclerosis [5] and systolic cardiac dysfunction [6, 7], as well as systemic metabolic disorders [8]. Endothelial cells (ECs) maintain vascular homeostasis, and healthy ECs respond to physical and chemical stimuli by producing various factors involved in regulation of vascular tone, cell adhesion, thrombosis, smooth muscle cell proliferation, and inflammation [9]. Risk factors for cardiovascular disease, including hypertension, obesity, diabetes, and aging, induce EC senescence mainly via activation of p53 signaling [8, 10–12]. Senescent endothelial cells have been detected in atherosclerotic plaque. Such senescent cells produce pro-inflammatory cytokines and promote the development of low grade sterile inflammation and tissue remodeling, contributing to increased susceptibility to atherosclerotic diseases.

Nitric oxide (NO) is mainly produced by endothelial nitric oxide synthase (eNOS) and it has a crucial role in maintaining EC homeostasis. It is known that EC senescence induces uncoupling of eNOS, leading to an increase of eNOS monomer levels and reduced NO activity [13–15]. Elevation of eNOS monomer levels promotes excessive production of ROS along with reduced NO production, further accelerating EC dysfunction.

Polyphenols are compounds found in many fruits, vegetables and beverages, including tea or wine [16]. A diet rich in polyphenols was reported to reduce cardiovascular disease [17], and high polyphenol intake decreased the mortality of subjects with cardiovascular risk factors compared to those with low polyphenol intake [18]. In addition, polyphenols are widely accepted to suppress vascular changes associated with aging [19–21]. Berries are rich in polyphenols, and eating berries was reported to reduce cardiovascular risk [22]. Boysenberries, also described as hybrid *Rubus* berry of *Rubus baileyanus* and *Rubus loganobaccus*, are an abundant source of polyphenols that may have various physiological effects. In fact, boysenberry juice was reported to lower the blood pressure of spontaneously hypertensive rats [23], and improve a flow-mediated dilation (FMD) of the brachial artery [24]. There are four types of polyphenols, including phenolic acids, flavonoids, stilbenes, and lignans. Flavonoids are further classified into six subclasses (flavonols, flavones, isoflavones, flavanones, flavonols and anthocyanidins). Anthocyanins (AC) are natural water soluble pigments derived from anthocyanidins by addition of sugars. AC are known to have a vascular protective effect by suppressing production of ROS and activating NO production. AC were also reported to block the expression of adhesion molecules of vessels. Furthermore, higher intake of AC is associated with a lower risk of myocardial infarction [25], and AC isolated from berries (320 mg/day) improve endothelium-dependent vasodilatation in hypercholesterolemic patients [26]. While these studies have indicated a protective effect of polyphenols and their components against vascular pathology, the underlying mechanisms are yet to be defined. Here, we show that boysenberry polyphenol (BP) and AC (the main component of BP) inhibit endothelial dysfunction and improve vascular function under metabolic stress.

## Materials and methods

### Boysenberry polyphenol extraction

Boysenberry juice was obtained from Berryfruit Export New Zealand Ltd. (New Zealand). Extracted ingredients of polyphenols were referred to the previous reports [27, 28]. Boysenberry juice was loaded to a Amberlite XAD-7HP column (Organo, Japan), and the column was washed with water, and eluted with ethanol. The eluate was evaporated in vacuo and powdered as reported previously [27]. The powder was named boysenberry polyphenol (BP). BP was dissolved in ethanol:water:formic acid (80:20:1). The solution was loaded to a Dowex 50W-X8 column (Dow Chemical, US). The column was washed with ethanol:water:formic acid (80:20:1), and anthocyanin fraction (AC) was eluted with ethanol:water:hydrochloric acid (50:50:10) as reported previously [28].

### HPLC analysis

Anthocyanins in BP and AC was analyzed by Shimadzu Prominence UFLC system (Shimadzu Corp., Kyoto, Japan) equipped with a Photodiode Array Detector and 4.6mm i.d. x 250mm of Inertsil ODS-3 (GL sciences, Japan, 5020–01732) as previously reported [27]. Anthocyanins were separated by 0.5% trifluoroacetic acid/water to acetonitrile at 40°C and detected at 520 nm, and quantified with standard product of cyanidin-3-glucoside (Extrasynthese, France, 0915S).

### Animal model

All animal experiments were conducted in compliance with the protocol reviewed and approved by the Institutional Animal Care and Use Committee of Niigata University. C57BL/6NCr mice were purchased from SLC (Japan). Mice were kept under standard housing with free access to food and water at room temperature (23–24°C) in a 12hr light-dark cycle. Mice were fed High Fat Diet 32 (CLEA Japan) in the high fat diet (HFD) group or CE-2 (CLEA Japan) in the normal chow (NC) group from 4 weeks of age. From 12 weeks, HFD mice received 0.1% BP in the drinking water. At 18–20 weeks, after the animals were euthanized by 100mg/kg intraperitoneal pentobarbital (Kyoritsu Seiyaku Co., japan, Somnopentyl^®^) injection, tissues were quickly corrected and vascular function was evaluated.

### Investigation of vascular reactivity

After mice were anesthetized, the iliac arteries were removed and placed into the organ bath of the Wire Myograph System (DMT, Denmark) to evaluate vascular reactivity, as described previously [27]. The vascular pressure was adjusted to 100 mmHg at 37°C, and equilibration was done with physiological saline solution (PSS) containing 118.99 mM NaCl, 4.69 mM KCl, 1.17 mM MgSO_4_, 25 mM NaHCO_3_, 2.5 mM CaCl_2_, 1.18 mM KH_2_PO_4_, 0.03 mM EDTA, and 5.5 mM glucose. PSS was aerated with 95% O2 and 5% CO_2_ and was maintained at pH 7.4. The vessels were stretched with 75 mM KCL solution and 10 μM noradrenaline. After equilibration, 10^−5^ M phenylephrine (PE) was added for pre-contraction, and 10^−9^ to 10^−5^ M acetylcholine (Ach) was cumulatively added to the organ bath after stabilization of the vascular response for evaluation of EC function. Subsequently, the vessels were washed with PSS and again pre-contracted with PE. L-NAME (3 × 10^−4^ M) was added to inhibit basal NO production. Then sodium nitroprusside was cumulatively added to the organ bath from 10^−9^ to 10^−5^ M for evaluation of smooth muscle function. Relaxation responses to Ach and SNP were calculated as the percent change of force (mN) by the following equation: percent dilation = 100% × (1 –(Force _after Ach/SNP_ –Force _basal_) / (Force _initial diameter after PE_ –Force _basal_)). To assess the effects of AC, iliac arteries were obtained from HFD mice and incubated with PSS containing AC for 6 hours in a CO_2_ incubator at 37°C, after which vascular reactivity was evaluated.

### Histological examination

Part of the descending aorta was immersed in OCT compound (Leica), and cut into 10 μm sections on a cryostat (Leica). NO production was evaluated by staining with diaminorhodamine-4M acetoxymethyl ester (DAR-4M, Goryo Chemical, Japan, SK1006-01) as previously reported [29]. Frozen sections were reacted with 10 μM DAR-4M in PSS for 1 hr at 37°C and then fixed with 4% paraformaldehyde (PFA). Then fluorescence was observed using a TRITC filter (Em: 545 nm, Ex: 605 nm) and the aortic fluorescence intensity was measured per field at x200 magnification. Production of reactive oxygen species (ROS) was evaluated by staining with dihydroethidium (DHE) as described previously with slight modification [30, 31]. Frozen sections were incubated with DHE (10 μmol/L) in PBS for 30 minutes at 37°C, after which the sections were observed under a TRITC filter and the aortic fluorescence intensity was measured per field at x200 magnification. For immunostaining, the primary antibodies were anti-p53 antibody (CM5) (Leica Biosystems, P53-CM5P-L), isolectin GS-IB4 from Griffonia simplicifolia biotin-XX conjugate (Invitrogen, I21414), and Hoechst 33258 (Thermo Fisher Scientific). The secondary antibodies were Cy5 Goat Anti-Rabbit IgG (H+L) (Life Technologies, A10523) for anti-p53 and BB515 streptavidin (BD horizon, 564453) for isolectin GS-IB4 from Griffonia simplicifolia biotin-XX conjugate. Antibodies were used at a concentration of 1:50, except for anti-p53 (1:500) and Hoechst 33258 (1:1000, Thermo Fisher Scientific, H21491). Images of the stained sections were obtained with a Biorevo (Keyence, Japan). Fluorescence signals were quantified with Image-J software at a magnification of x200 for NO and DHE in the aorta, x1200 for p53 in the aorta, and x400 for NO and DHE in HUVECs. Four fields were randomly selected in each section for quantification, except that NO and DHE signals in the aorta were analyzed in one field at a lower magnification.

### Cell culture

Human umbilical vein endothelial cells (HUVECs, Lonza) were cultured with Endothelial Cell Growth Medium-2 BulletKit (Lonza). For evaluation of NO production, HUVECs were incubated with 500 μM palmitic acid and 10 μg/ml BP or 10 μg/ml AC in FBS-free medium for 6 hr. Then the medium was exchanged for FBS-free medium with 5μM DAR-4M and incubation was continued for 30 min. Subsequently, 100 nM insulin was added and the HUVECs were incubated for a further 30 min, after which the cells were fixed with 4% PFA and observed. For evaluation of ROS production, HUVECs were pre-incubated with 10 μg/ml BP or 10 μg/ml AC for 2 hr, after which 500 μM palmitic acid was added and the cells were incubated for 15 min. Subsequently, the HUVECs were fixed with 4% PFA and incubated with DHE for 30 min at 37°C. Stock solution of palmitic acid (5 mM) was prepared by conjugation with 10% BSA.

### Western blot analysis

Protein lysates were prepared in RIPA lysis buffer with protease inhibitor cocktail (Roche), lysates were separated by SDS-PAGE, and the proteins were transferred to a PVDF membrane (Millipore). The membrane was reacted with the following primary antibodies at 1:1000: anti-p53 antibody (DO1) (Santa Cruz, sc-126), anti-eNOS (BD Transduction, 610296), anti-phospho-eNOS (ser1177) (Cell Signaling Technology, CST9571), anti-β-actin (Cell Signaling Technology, 4970), and anti-α-tubulin (Cell Signaling Technology, 2125). Blocking was done with 5% skim milk. The secondary antibodies were horseradish peroxidase-conjugated anti-mouse immunoglobulin-G (Jackson, 115-035-003) for anti-p53 and anti-eNOS, as well as horseradish peroxidase-conjugated anti-rabbit immunoglobulin-G (Jackson, 115-035-144) for anti-phospho-eNOS, anti-β-actin and anti-α-tubulin. Secondary antibodies were added at a concentration of 1:5000. Proteins were detected by chemiluminescence using ECL western blotting detection reagents (GE Healthcare, UK). Relative protein levels were quantified with Image J software and were normalized by using β-actin or α-tubulin as loading controls.

### Hemodynamic measurements and laboratory tests

A tail-cuff system (BP-98A, Softron Co., Tokyo, Japan) was used to measure arterial blood pressure (BP) and heart rate (HR) when mice became 8 weeks of age. Intraperitoneal glucose tolerance test (IGTT) was done as previously described with slight modification and glucose was intraperitoneally given at a dose of 1g kg^-1^(body weight) [32].

### Low-temperature SDS-PAGE

eNOS dimer and monomer were measured by low-temperature SDS-PAGE [33]. Protein lysates were mixed with SDS sample buffer containing mercaptoethanol at 4°C and samples were separated by SDS-PAGE at 4°C. Then eNOS protein was detected by western blot analysis.

### RNA analysis

Total RNA (1 μg) was isolated from tissue samples with RNA-Bee (TEL-TEST Inc.). Real-time PCR (qPCR) was performed by using a Light Cycler 480 (Roche) with the Universal Probe Library and the Light Cycler 480 Probes Master (Roche) according to the manufacturer’s instructions. The primers and their sequences were as follows. *Actb*, was used as the internal control.

*Cdkn1a*; 5’-tccacagcgatatccagaca-3’, 5’-ggacatcaccaggattggac-3’;

*Actb*; 5’-ctaaggccaaccgtgaaaag-3, 5’-accagaggcatacagggaca-3’.

### Statistical analysis

Data are shown as the mean ± SEM. Differences between groups were examined by Student’s t-test or two-way ANOVA, followed by Tukey’s multiple comparison test. Some studies are analyzed with repeated measures followed by Tukey’s multiple comparison test. For all analyses, p< 0.05 was considered statistically significant.

## Results

### Boysenberry polyphenol inhibits endothelial dysfunction and improves vascular function in obese mice

**Fig 1:**
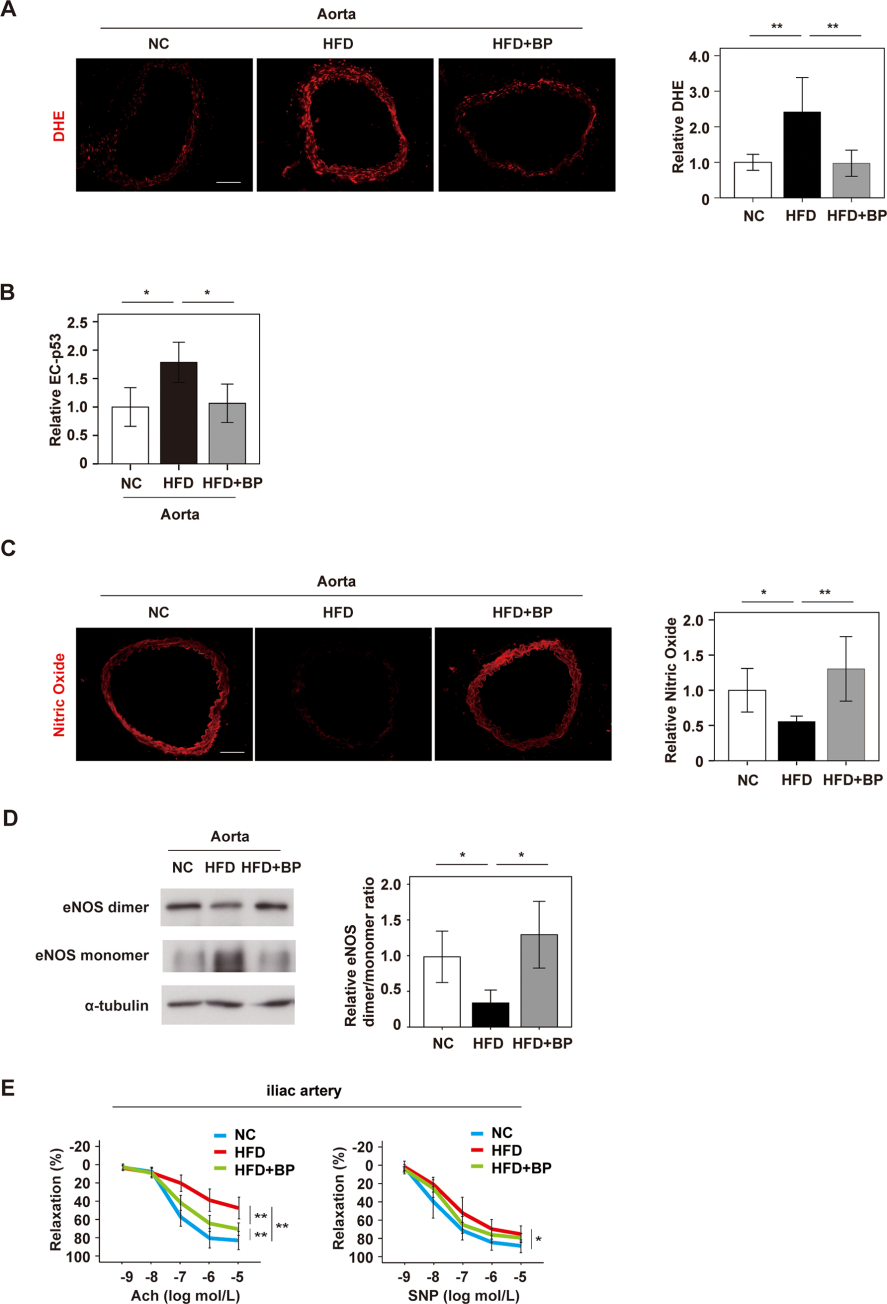
Boysenberry polyphenol inhibits endothelial dysfunction and improves vascular function in obese mice. Wild-type mice were fed normal chow (NC) or a high fat diet (HFD). In some groups, boysenberry polyphenol (BP; 0.1% in the drinking water) was administrated in addition to the HFD (HFD+BP). **A.** DHE staining of aortas from NC, HFD, and HFD+BP mice (Scale bar = 100 μm). The right graph shows the relative fluorescence intensity of DHE (n = 8, 9, and 9). **B.** Immunofluorescence staining and quantification of relative p53 expression by EC in the aorta (n = 6, 6, and 6). **C.** DAR-4M staining of the aorta to detect nitric oxide (Scale bar = 100 μm). The right graph shows the relative fluorescence intensity (n = 9, 9, and 10). **D.** Western blot analysis of eNOS dimer, eNOS monomer, and α-tubulin in the aorta. The right panel shows quantification of the eNOS dimer/monomer ratio adjusted for α-tubulin (n = 8, 7, and 8). **E.** The left panel indicates endothelium-dependent relaxation of iliac arteries in response to escalating doses of acetylcholine (Ach). The right panel indicates endothelium-independent relaxation of iliac arteries in response to escalating doses of sodium nitroprusside (SNP) (n = 6, 8, and 8). Data were analyzed by the 2-tailed Student’s t-test (C), 2-way ANOVA (A, B and D), followed by Tukey’s multiple comparison test, or repeated measures followed by Tukey’s multiple comparison test (E). *P < 0.05; **P < 0.01. Values represent the mean ± SEM.

It was previously reported that dietary obesity induces p53-mediated EC senescence, which in turn promotes systemic metabolic dysfunction [8]. However, the influence of EC senescence on vascular function under metabolic stress was not clearly determined in previous studies, and this promoted us to investigate the role of EC senescence in vascular homeostasis. We generated wild-type mice with dietary obesity on a C57BL6/NCr background by maintaining the animals on a high fat diet (HFD). In HFD mice, administration of BP caused no significant changes of body weight, food intake, systolic or diastolic blood pressure, heart rate, and systemic glucose intolerance (Parts A–C in S1 Fig). Metabolic stress due to obesity increased the ROS level in the aorta, and this change was ameliorated by BP (Fig 1A). Immunofluorescence studies showed a significant increase of endothelial p53 expression with dietary obesity, which was suppressed by administration of BP (Fig 1B). One of the p53 target genes, *Cdkn1a*, was also shown to reduce in aorta by BP treatment (Part D in S1 Fig). The nitric oxide (NO) level was reduced in the aortas of HFD mice, while this change was ameliorated by BP (Fig 1C). Endothelial NO synthase (eNOS) has pivotal role in balancing the production of NO and ROS. Dimerized eNOS produces NO. However, monomerization (uncoupling) of eNOS occurs with stress, and eNOS monomer mediates ROS production. HFD mice showed a marked decrease of the eNOS dimer/monomer ratio in the aorta, while this change was ameliorated by BP administration (Fig 1D). Total eNOS level was comparable between HFD and HFD+BP group (Part E in S1 Fig). These results suggested that BP maintains vascular homeostasis through suppression of eNOS uncoupling induced by metabolic stress, as well as by increasing the bioavailability of NO. Next, we evaluated vascular reactivity by using a wire myograph system. HFD mice showed a marked reduction of endothelium-dependent vasodilatation in the iliac artery, which was significantly restored by BP administration (Fig 1E). The vascular response to sodium nitroprusside was significantly attenuated in HFD mice and was not significantly improved by BP treatment, suggesting that BP has little influence on endothelium-independent vasodilation (Fig 1E). These results indicated that BP had preventive effect on endothelial dysfunction induced by metabolic stress.

### Boysenberry polyphenol inhibits endothelial dysfunction induced by metabolic stress in vitro

**Fig 2:**
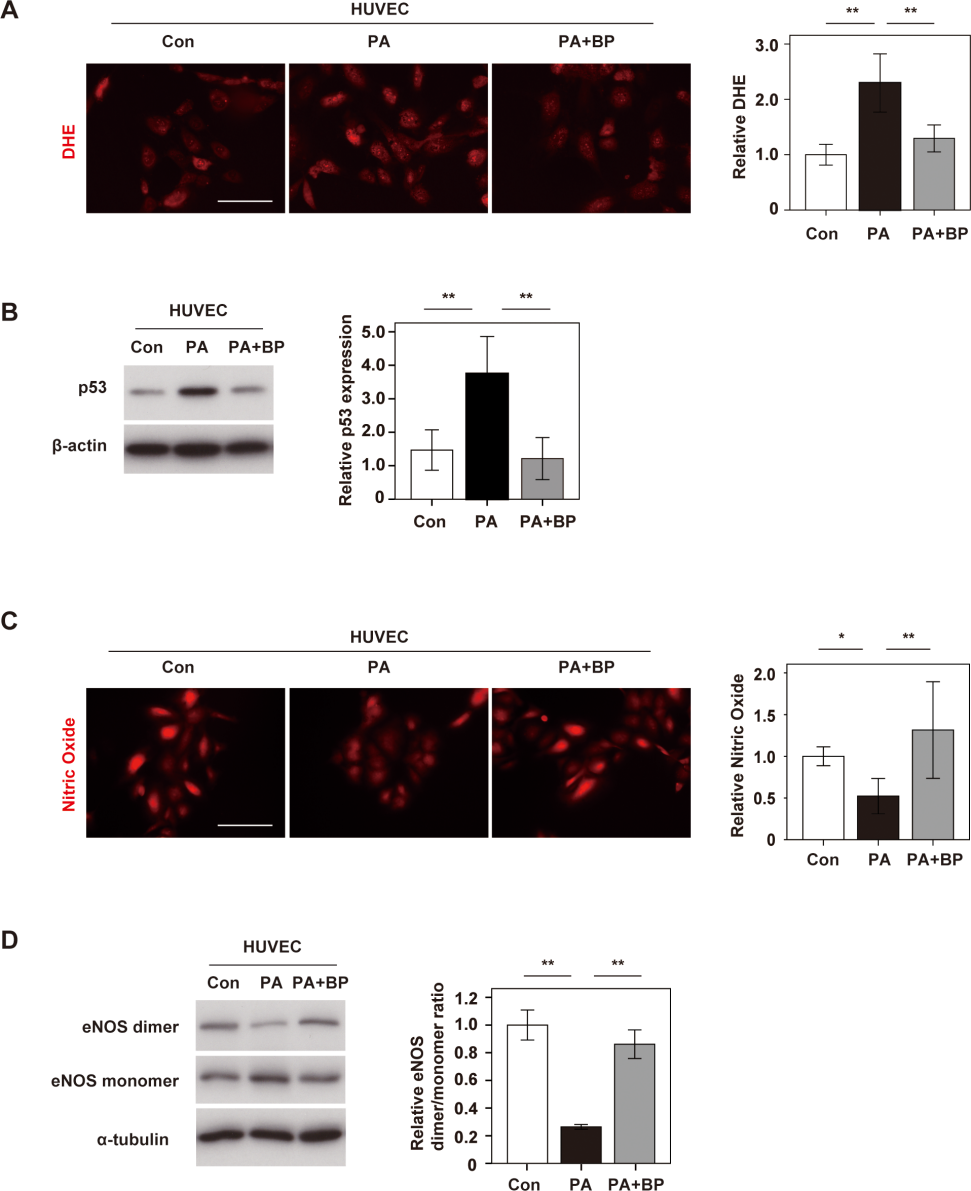
Boysenberry polyphenol inhibits metabolic stress-induced endothelial dysfunction. Human umbilical vein endothelial cells (HUVECs) were treated with BSA (Con group), palmitic acid (500 μM) (PA group(6hr)), or PA (500 μM) + BP (10 μg/ml) (PA+BP group). **A.** DHE staining of HUVECs in Con, PA, and PA+BP groups (Scale bar = 100 μm). The right graph shows the relative fluorescence intensity of DHE (n = 4, 4, and 4). **B.** Western blot analysis of p53 expression in HUVECs. The right panel displays quantification of p53 relative to the β-actin loading control (n = 6, 6, and 6). **C.** DAR-4M staining of HUVECs to detect nitric oxide (Scale bar = 100 μm). The right graph shows the relative fluorescence intensity (n = 4, 4, and 4). **D.** Western blot analysis of eNOS dimer, eNOS monomer, and α-tubulin in HUVECs. The right panel shows quantification of the eNOS dimer/ monomer ratio adjusted for α-tubulin (n = 3, 3, and 3). Data were analyzed by 2-way ANOVA, followed by Tukey’s multiple comparison test. *P < 0.05; **P < 0.01. Values represent the mean ± SEM.

To further investigate the role of BP in EC homeostasis, we performed in vitro studies that assessed the inhibitory effect of BP on endothelial dysfunction. When human umbilical vein endothelial cells (HUVECs) were cultured with palmitic acid, there was a marked increase of ROS, while this response was significantly suppressed by treatment with BP (Fig 2A). Palmitic acid also increased p53 expression in HUVECs, while BP suppressed it, suggesting that BP inhibited metabolic stress-induced endothelial dysfunction (Fig 2B). Next, we evaluated the effect on NO production. Palmitic acid reduced NO production by HUVECs, both in short (6hr) and chronic (1week) phase, and this effect was ameliorated with administration of BP (Fig 2C and Part A in S2 Fig). Finally, we examined eNOS uncoupling in HUVECs. Palmitic acid promoted eNOS monomerization, while this change was suppressed by BP (Fig 2D). Palmitic acid led to a significant reduction in phospho-eNOS level, and this reduction was significantly ameliorated with BP administration (Part B in S2 Fig). These results suggested that BP also promoted EC homeostasis in the in vitro setting.

### Anthocyanins purified from boysenberry polyphenol protect endothelial cells

**Fig 3:**
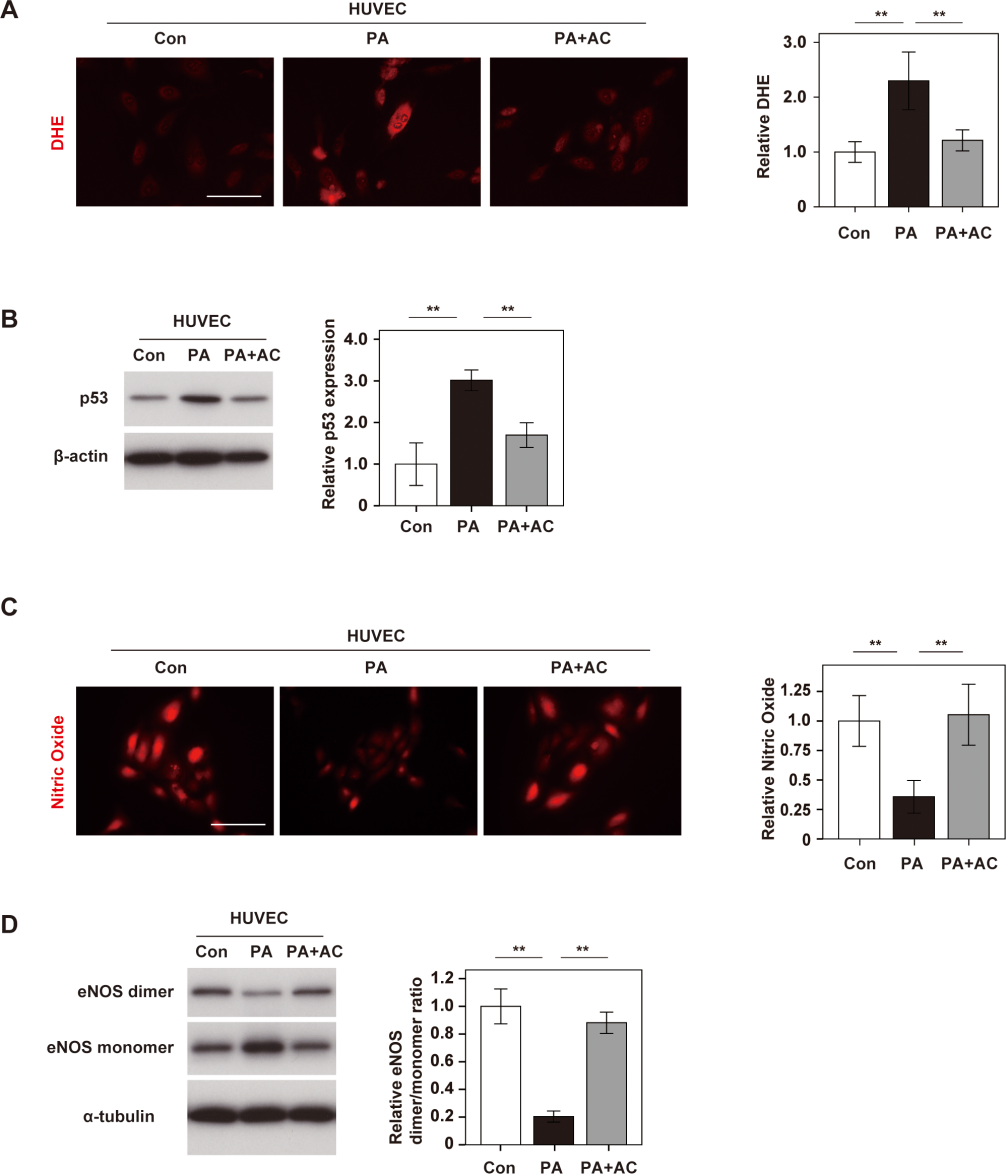
Anthocyanins purified from boysenberry polyphenol protect endothelial cells. Human umbilical vein endothelial cells (HUVECs) were treated with BSA (Con group), palmitic acid (500 μM) (PA group), or PA (500 μM) + anthocyanins (AC) (10 μg/ml) (PA+AC group). **A.** DHE staining of HUVECs in Con, PA, and PA+AC groups (Scale bar = 100 μm). The right graph shows the relative fluorescence intensity of DHE (n = 4, 4, and 4). **B.** Western blot analysis of p53 expression in HUVECs. The right panel displays quantification of p53 relative to the β-actin loading control (n = 3, 3, and 3). **C.** DAR-4M staining of HUVECs for nitric oxide (Scale bar = 100 μm). The right graph shows the relative fluorescence intensity (n = 4, 4, and 4). **D.** Western blot analysis of eNOS dimer, eNOS monomer, and α-tubulin expression in HUVECs. The right panel displays quantification of the eNOS dimer/ monomer ratio adjusted for α-tubulin (n = 3, 3, and 3). Data were analyzed by 2-way ANOVA, followed by Tukey’s multiple comparison test. *P < 0.05; **P < 0.01. Values represent the mean ± SEM.

In order to identify the component of BP mainly contributing to vascular endothelial protection, we focused on anthocyanins (AC), which are known to be a major constituent of BP. AC include cyanidin-3-glucoside, cyanidin-3- [2-(glucosyl)glucoside], cyanidin-3-[2-(glucosyl)-6-(rhamnosyl)glucoside], and cyanidin-3-[6-(rhamnosyl)glucoside] [27], and it was reported that these compounds have a protective effect against cardiovascular disorders. AC was extracted from BP as previously described [27] (Parts A and B in S3 Fig), and we found AC administration inhibited palmitic acid-induced ROS production in HUVECs (Fig 3A), and also inhibited the increase of p53 expression in palmitic acid-treated HUVECs (Fig 3B). In addition, inhibition of NO production and eNOS uncoupling in HUVECs under metabolic stress were ameliorated by AC (Fig 3C and 3D). These results suggested that AC had an important role in mediation of EC protection by BP.

### Anthocyanins purified from boysenberry polyphenol improve vascular function

**Fig 4:**
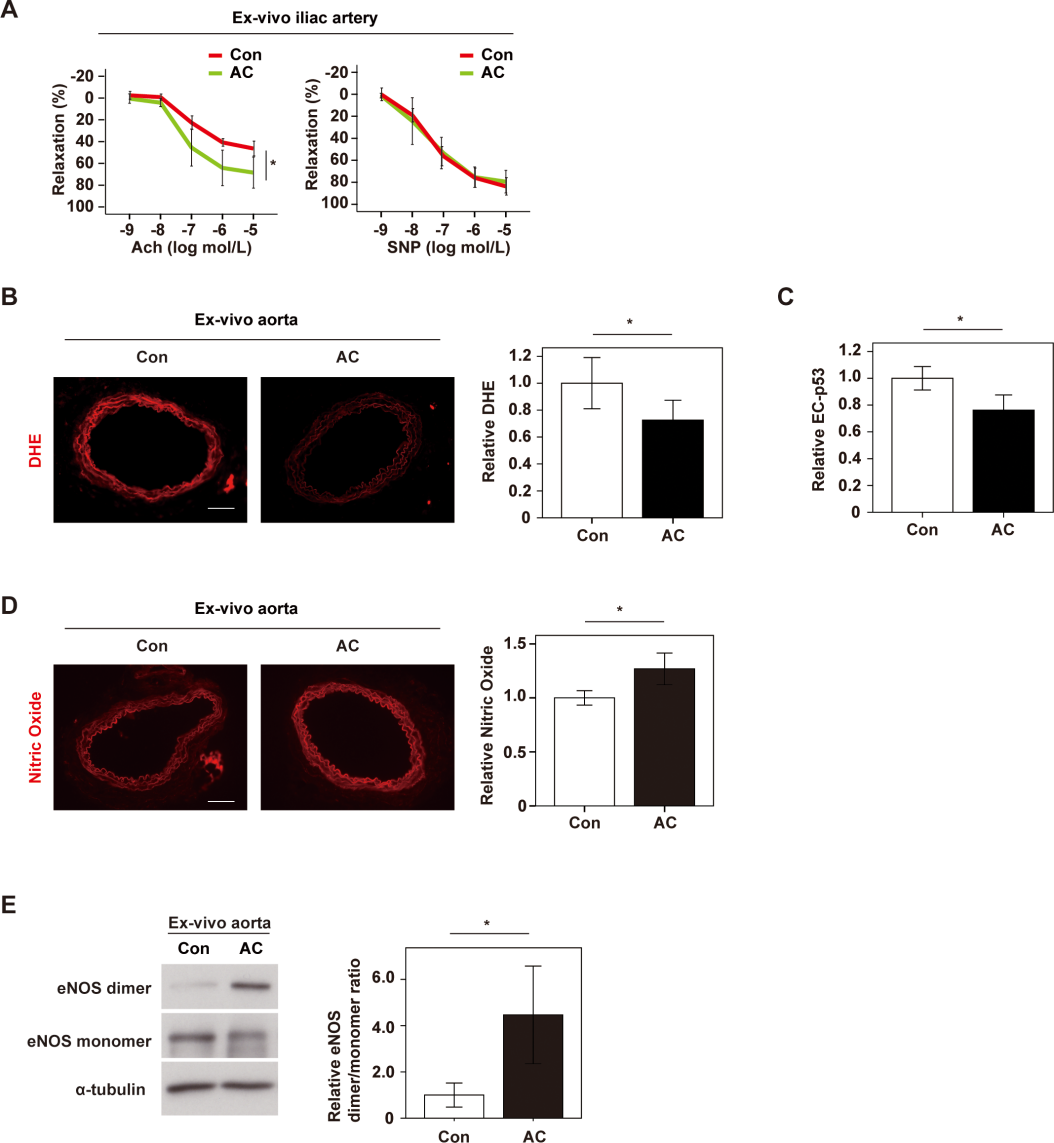
Anthocyanins purified from boysenberry polyphenol improve vascular function. Iliac arteries or aortas extracted from mice with dietary obesity were incubated ex vivo with PBS (Con group) or anthocyanins (AC group) for 6 hours, and the following studies were performed. **A.** The left panel shows endothelium-dependent relaxation of iliac arteries in response to escalating doses of acetylcholine (Ach). The right panel indicates endothelium-independent relaxation of iliac arteries in response to escalating doses of sodium nitroprusside (SNP) (n = 4 and 4). **B.** DHE staining of ex vivo-incubated aortas in Con and AC groups (Scale bar = 100 μm). The right graph shows the relative fluorescence intensity of DHE (n = 8 and 8). **C.** Immunofluorescence staining for quantification of relative p53 expression in EC in the aorta (n = 5 and 5). **D.** DAR-4M staining of ex vivo-incubated aortas to detect nitric oxide (Scale bar = 100 μm). The right graph shows the relative fluorescence intensity (n = 6 and 6). **E.** Western blot analysis of eNOS dimer, eNOS monomer and α-tubulin in ex vivo incubated aortas. The right panel displays quantification of the eNOS dimer/ monomer ratio adjusted for α-tubulin (n = 5 and 6). Data were analyzed by the 2-tailed Student’s t-test (B-E) or repeated measures (A). *P < 0.05; **P < 0.01. Values represent the mean ± SEM.

To further evaluate the effects of AC on vascular function, we performed ex vivo studies on iliac arteries obtained from mice with dietary obesity. We found that AC significantly improved endothelium-dependent vasodilatation of iliac arteries, but had little influence on the response to sodium nitroprusside (Fig 4A). Also, ex vivo addition of AC to aortic specimens suppressed ROS production and reduced expression of p53 by EC (Fig 4B and 4C). We also found that AC increased NO production, in association with suppression of eNOS uncoupling (Fig 4D and 4E). Taken together, these findings suggested that AC were the chief component of BP mediating EC protection against metabolic stress. Our results indicated that BP and AC promoted vascular homeostasis under metabolic stress by suppressing endothelial dysfunction and increasing the bioavailability of NO.

## Discussion

In this study, we showed that BP could inhibit endothelial dysfunction under metabolic stress. AC are a major constituent of BP, and we found that AC purified from BP suppressed p53 expression and ROS production. These effects were associated with suppression of eNOS uncoupling and elevation of NO bioavailability, suggesting that AC may be the main factor mediating the biological effects of BP. Various foods that can be consumed on a daily basis contain AC, including grapes and berries [34]. AC have been reported to have favorable effects on cardiovascular function [35–37]. AC were shown to inhibit atherosclerosis in apolipoprotein E (ApoE)-deficient mice [38], and AC reduced the risk of myocardial infarction in another study [25]. Furthermore, intake of AC isolated from berries was reported to improve flow-mediated dilation (FMD) in humans [26, 39]. These reports suggest that AC could make an important contribution to reducing cardiovascular risk.

Boysenberry juice has a high content of AC. Intake of boysenberry juice was reported to reduce the blood pressure in spontaneously hypertensive rats (SHR) [23] and improve FMD in humans [24]. In the aortas of our mice with dietary obesity and in HUVECs, BP attenuated ROS production by ECs and also inhibited p53 expression. It is well known that excessive exposure to ROS results in accumulation of DNA damage and induces cellular senescence, mainly via p53/p21 signaling [40]. AC were reported to induce activation of nuclear factor erythroid 2-related factor 2 (Nrf2), which has a critical role in induction of antioxidant proteins [41–43]. AC also induce the expression of antioxidant enzymes, such as heme oxygenase-1 (HO-1) [44] and glutathione [44], as well as activating catalase, peroxidase, and superoxide dismutase [45]. Furthermore, AC have been reported to inhibit NADPH oxidase activity [46, 47]. Moreover, AC can inhibit p53-induced EC senescence mediated by TNF-α [48] and UV exposure [49]. In line with these results, we demonstrated that AC purified from BP could suppress ROS production and inhibit p53 expression by EC under metabolic stress.

Dietary obesity was reported to reduce the dimerization of eNOS and increase its monomerization [50]. Uncoupling (monomerization) of eNOS suppresses NO production and also promotes an increase of ROS production. Several polyphenols are known to inhibit eNOS uncoupling. Resveratrol is a polyphenol that has been extensively studied and it has been reported to inhibit eNOS uncoupling in aged rats [51], SHR [52], and ApoE KO mice [53]. Uncoupling of eNOS occurs with deficiency of the NOS cofactor tetrahydrobiopterin (BH4), and resveratrol activates GTP cyclohydrolase 1 (GCH1), a rate-limiting enzyme in BH4 biosynthesis, leading to elevation of the BH4 level. We found that BP inhibited uncoupling of eNOS caused by exposure to an HFD or palmitic acid, resulting in increased bioavailability of NO in both the in vivo and in vitro settings. Endothelium-dependent vasodilatation showed a marked reduction in the iliac arteries of HFD mice, and this was significantly ameliorated by BP administration. In addition, we found that AC also suppressed eNOS uncoupling, increased NO production, and increased endothelium-dependent vasodilatation. Taken together, our results indicate that intake of BP or AC could potentially prevent vascular disorder by inhibiting endothelial dysfunction, thus contributing to suppression of cardiovascular disease.

## Supporting information

S1 Fig Baseline characteristics of mice administrated with Boysenberry polyphenol. Wild-type mice were fed normal chow (NC) or a high fat diet (HFD). In some groups, boysenberry polyphenol (BP; 0.1% in the drinking water) was administrated in addition to the HFD (HFD+BP). **A, B.** Body weight (n = 6,8,8) and Food intake(n = 4,6,6)(A), systolic blood pressure, diastolic blood pressure, and heart rate(B) of indicated mice group(n = 6,6,7). **C.** Glucose tolerance test of indicated mice group(n = 6,6,7). **D.** Transcript for *Cdkn1a* as analyzed in aorta from indicated mice group(n = 5,5,5). **E.** Western blot analysis of total eNOS, and β-actin in the aorta. The right panel shows quantification of the total eNOS adjusted for β-actin(n = 13,13). Data were analyzed by the 2-tailed Student’s t-test (E), 2-way ANOVA followed by Tukey’s multiple comparison test (A, B, D), or Repeated measures followed by Tukey’s multiple comparison test(C). *P < 0.05; **P < 0.01. Values represent the mean ± SEM.
(DOCX)

S2 Fig Boysenberry polyphenol improves nitric oxide production in HUVECs also in chronic phase. Human umbilical vein endothelial cells (HUVECs) were treated with BSA (Con group), palmitic acid (200μM (for 1week culture) or 500μM (for 6hr culture)) (PA group), or PA (200μM (for 1week culture) or 500μM (for 6hr culture)) + BP (10 μg/ml) (PA+BP group). HUVECs were cultured with PA and/or BP for totally 1week for studies in Part A in S2 Fig and 6hrs for studies in Part B in S2 Fig. **A.** DAR-4M staining of HUVECs to detect nitric oxide (Scale bar = 100 μm). The right graph shows the relative fluorescence intensity (n = 5,5,5). **B.** Western blot analysis of phospho-eNOS(p-eNOS), eNOS and β-actin in HUVECs. The right panel shows quantification of the p-eNOS adjusted for eNOS (n = 4,4,4,4). Data were analyzed by 2-way ANOVA followed by Tukey’s multiple comparison test(A), or the 2-tailed Student’s t-test (B). *P < 0.05; **P < 0.01. Values represent the mean ± SEM.
(DOCX)

S3 Fig Characterize of boysenberry polyphenols and anthocyanin fraction. A. High-performance liquid chromatography (HPLC) profile at 520 nm of anthocyanins (AC) from purified boysenberry polyphenol (BP). B. Classified anthocyanins (AC) were quantified using a cyanidin-3-glucoside standard.
(DOCX)

## References

[1] Hayflick L, Moorhead PS. The serial cultivation of human diploid cell strains. Exp Cell Res. 1961;25:585–621. . 13905658 10.1016/0014-4827(61)90192-6

[2] Tchkonia T, Zhu Y, van Deursen J, Campisi J, Kirkland JL. Cellular senescence and the senescent secretory phenotype: therapeutic opportunities. J Clin Invest. 2013;123(3):966–72. Epub 2013/03/05. 10.1172/JCI64098 ; PubMed Central PMCID: PMCPMC3582125. 23454759 PMC3582125

[3] Ghebre YT, Yakubov E, Wong WT, Krishnamurthy P, Sayed N, Sikora AG, et al Vascular Aging: Implications for Cardiovascular Disease and Therapy. Transl Med (Sunnyvale). 2016;6(4). Epub 2017/09/22. 10.4172/2161-1025.1000183 ; PubMed Central PMCID: PMC5602592. 28932625 PMC5602592

[4] Costantino S, Paneni F, Cosentino F. Ageing, metabolism and cardiovascular disease. J Physiol. 2016;594(8):2061–73. Epub 2015/09/24. 10.1113/JP270538 ; PubMed Central PMCID: PMC4933114. 26391109 PMC4933114

[5] Wang JC, Bennett M. Aging and atherosclerosis: mechanisms, functional consequences, and potential therapeutics for cellular senescence. Circ Res. 2012;111(2):245–59. Epub 2012/07/10. 10.1161/CIRCRESAHA.111.261388 . 22773427

[6] Yoshida Y, Shimizu I, Katsuumi G, Jiao S, Suda M, Hayashi Y, et al p53-Induced inflammation exacerbates cardiac dysfunction during pressure overload. J Mol Cell Cardiol. 2015;85:183–98. 10.1016/j.yjmcc.2015.06.001 . 26055447

[7] Gogiraju R, Xu X, Bochenek ML, Steinbrecher JH, Lehnart SE, Wenzel P, et al Endothelial p53 deletion improves angiogenesis and prevents cardiac fibrosis and heart failure induced by pressure overload in mice. J Am Heart Assoc. 2015;4(2). Epub 2015/02/26. 10.1161/JAHA.115.001770 ; PubMed Central PMCID: PMCPMC4345879. 25713289 PMC4345879

[8] Yokoyama M, Okada S, Nakagomi A, Moriya J, Shimizu I, Nojima A, et al Inhibition of endothelial p53 improves metabolic abnormalities related to dietary obesity. Cell Rep. 2014;7(5):1691–703. Epub 2014/05/27. 10.1016/j.celrep.2014.04.046 . 24857662

[9] Deanfield JE, Halcox JP, Rabelink TJ. Endothelial function and dysfunction: testing and clinical relevance. Circulation. 2007;115(10):1285–95. Epub 2007/03/14. 10.1161/CIRCULATIONAHA.106.652859 . 17353456

[10] Minamino T, Komuro I. Vascular cell senescence: contribution to atherosclerosis. Circ Res. 2007;100(1):15–26. Epub 2007/01/06. 10.1161/01.RES.0000256837.40544.4a . 17204661

[11] Brodsky SV, Gealekman O, Chen J, Zhang F, Togashi N, Crabtree M, et al Prevention and reversal of premature endothelial cell senescence and vasculopathy in obesity-induced diabetes by ebselen. Circ Res. 2004;94(3):377–84. Epub 2003/12/13. 10.1161/01.RES.0000111802.09964.EF . 14670841

[12] Minamino T, Orimo M, Shimizu I, Kunieda T, Yokoyama M, Ito T, et al A crucial role for adipose tissue p53 in the regulation of insulin resistance. Nat Med. 2009;15(9):1082–7. Epub 2009/09/01. 10.1038/nm.2014 . 19718037

[13] Sena CM, Pereira AM, Seica R. Endothelial dysfunction—a major mediator of diabetic vascular disease. Biochim Biophys Acta. 2013;1832(12):2216–31. Epub 2013/09/03. 10.1016/j.bbadis.2013.08.006 . 23994612

[14] Landmesser U, Dikalov S, Price SR, McCann L, Fukai T, Holland SM, et al Oxidation of tetrahydrobiopterin leads to uncoupling of endothelial cell nitric oxide synthase in hypertension. J Clin Invest. 2003;111(8):1201–9. 10.1172/JCI14172 ; PubMed Central PMCID: PMCPMC152929. 12697739 PMC152929

[15] Yang YM, Huang A, Kaley G, Sun D. eNOS uncoupling and endothelial dysfunction in aged vessels. Am J Physiol Heart Circ Physiol. 2009;297(5):H1829–36. 10.1152/ajpheart.00230.2009 ; PubMed Central PMCID: PMCPMC2781386. 19767531 PMC2781386

[16] Manach C, Scalbert A, Morand C, Remesy C, Jimenez L. Polyphenols: food sources and bioavailability. Am J Clin Nutr. 2004;79(5):727–47. 10.1093/ajcn/79.5.727 . 15113710

[17] Tresserra-Rimbau A, Rimm EB, Medina-Remon A, Martinez-Gonzalez MA, de la Torre R, Corella D, et al Inverse association between habitual polyphenol intake and incidence of cardiovascular events in the PREDIMED study. Nutr Metab Cardiovasc Dis. 2014;24(6):639–47. 10.1016/j.numecd.2013.12.014 . 24552647

[18] Tresserra-Rimbau A, Rimm EB, Medina-Remon A, Martinez-Gonzalez MA, Lopez-Sabater MC, Covas MI, et al Polyphenol intake and mortality risk: a re-analysis of the PREDIMED trial. BMC Med. 2014;12:77 10.1186/1741-7015-12-77 ; PubMed Central PMCID: PMCPMC4102266. 24886552 PMC4102266

[19] Khurana S, Venkataraman K, Hollingsworth A, Piche M, Tai TC. Polyphenols: benefits to the cardiovascular system in health and in aging. Nutrients. 2013;5(10):3779–827. 10.3390/nu5103779 ; PubMed Central PMCID: PMCPMC3820045. 24077237 PMC3820045

[20] Santilli F, D'Ardes D, Davi G. Oxidative stress in chronic vascular disease: From prediction to prevention. Vasc Pharmacol. 2015;74:23–37. 10.1016/j.vph.2015.09.003 PubMed PMID: WOS:000366146100003. 26363473

[21] Suganya N, Bhakkiyalakshmi E, Sarada DV, Ramkumar KM. Reversibility of endothelial dysfunction in diabetes: role of polyphenols. Br J Nutr. 2016;116(2):223–46. 10.1017/S0007114516001884 . 27264638

[22] Huang H, Chen G, Liao D, Zhu Y, Xue X. Effects of Berries Consumption on Cardiovascular Risk Factors: A Meta-analysis with Trial Sequential Analysis of Randomized Controlled Trials. Sci Rep. 2016;6:23625 10.1038/srep23625 ; PubMed Central PMCID: PMCPMC4804301. 27006201 PMC4804301

[23] Matsushima A, Furuuchi R, Shirai M, Nagai S, Yokoyama T, Nishida H, et al Effects of acute and chronic boysenberry intake on blood pressure and endothelial function in spontaneous hypertensive rats. J Nutr Sci Vitaminol (Tokyo). 2014;60(1):43–51. . 24759259 10.3177/jnsv.60.43

[24] Matsusima A, Furuuchi R, Sakaguchi Y, Goto H, Yokoyama T, Nishida H, et al Acute and chronic flow-mediated dilation and blood pressure responses to daily intake of boysenberry juice: a preliminary study. Int J Food Sci Nutr. 2013;64(8):988–92. 10.3109/09637486.2013.812617 . 23848379

[25] Cassidy A, Mukamal KJ, Liu L, Franz M, Eliassen AH, Rimm EB. High anthocyanin intake is associated with a reduced risk of myocardial infarction in young and middle-aged women. Circulation. 2013;127(2):188–96. 10.1161/CIRCULATIONAHA.112.122408 ; PubMed Central PMCID: PMCPMC3762447. 23319811 PMC3762447

[26] Zhu Y, Xia M, Yang Y, Liu F, Li Z, Hao Y, et al Purified anthocyanin supplementation improves endothelial function via NO-cGMP activation in hypercholesterolemic individuals. Clin Chem. 2011;57(11):1524–33. 10.1373/clinchem.2011.167361 . 21926181

[27] Furuuchi R, Yokoyama T, Watanabe Y, Hirayama M. Identification and quantification of short oligomeric proanthocyanidins and other polyphenols in boysenberry seeds and juice. J Agric Food Chem. 2011;59(8):3738–46. 10.1021/jf104976n . 21391678

[28] Kool MM, Comeskey DJ, Cooney JM, McGhie TK. Structural identification of the main ellagitannins of a boysenberry (Rubus loganbaccus x baileyanus Britt.) extract by LC-ESI-MS/MS, MALDI-TOF-MS and NMR spectroscopy. Food Chem. 2010;119(4):1535–43. 10.1016/j.foodchem.2009.09.039 PubMed PMID: WOS:000272989600034.

[29] Nunez C, Victor VM, Tur R, Alvarez-Barrientos A, Moncada S, Esplugues JV, et al Discrepancies between nitroglycerin and NO-releasing drugs on mitochondrial oxygen consumption, vasoactivity, and the release of NO. Circ Res. 2005;97(10):1063–9. 10.1161/01.RES.0000190588.84680.34 PubMed PMID: WOS:000233173700016. 16224067

[30] Cheang WS, Ngai CY, Tam YY, Tian XY, Wong WT, Zhang Y, et al Black tea protects against hypertension-associated endothelial dysfunction through alleviation of endoplasmic reticulum stress. Sci Rep-Uk. 2015;5. doi: ARTN 10340 10.1038/srep10340 PubMed PMID: WOS:000355249600001. 25976123 PMC4432571

[31] Tronel C, Rochefort GY, Arlicot N, Bodard S, Chalon S, Antier D. Oxidative Stress Is Related to the Deleterious Effects of Heme Oxygenase-1 in an In Vivo Neuroinflammatory Rat Model. Oxid Med Cell Longev. 2013. doi: Artn 264935 10.1155/2013/264935 PubMed PMID: WOS:000316142700001. 23533686 PMC3606782

[32] Shimizu I, Yoshida Y, Moriya J, Nojima A, Uemura A, Kobayashi Y, et al Semaphorin3E-induced inflammation contributes to insulin resistance in dietary obesity. Cell Metab. 2013;18(4):491–504. 10.1016/j.cmet.2013.09.001 . 24093674

[33] Cortes-Gonzalez C, Barrera-Chimal J, Ibarra-Sanchez M, Gilbert M, Gamba G, Zentella A, et al Opposite effect of Hsp90alpha and Hsp90beta on eNOS ability to produce nitric oxide or superoxide anion in human embryonic kidney cells. Cell Physiol Biochem. 2010;26(4–5):657–68. 10.1159/000322333 . 21063103

[34] Wu X, Beecher GR, Holden JM, Haytowitz DB, Gebhardt SE, Prior RL. Concentrations of anthocyanins in common foods in the United States and estimation of normal consumption. J Agric Food Chem. 2006;54(11):4069–75. 10.1021/jf060300l . 16719536

[35] Cassidy A. Berry anthocyanin intake and cardiovascular health. Mol Aspects Med. 2017 10.1016/j.mam.2017.05.002 . 28483533

[36] Fairlie-Jones L, Davison K, Fromentin E, Hill AM. The Effect of Anthocyanin-Rich Foods or Extracts on Vascular Function in Adults: A Systematic Review and Meta-Analysis of Randomised Controlled Trials. Nutrients. 2017;9(8). 10.3390/nu9080908 ; PubMed Central PMCID: PMCPMC5579701. 28825651 PMC5579701

[37] de Pascual-Teresa S, Moreno DA, Garcia-Viguera C. Flavanols and anthocyanins in cardiovascular health: a review of current evidence. Int J Mol Sci. 2010;11(4):1679–703. 10.3390/ijms11041679 ; PubMed Central PMCID: PMCPMC2871133. 20480037 PMC2871133

[38] Miyazaki K, Makino K, Iwadate E, Deguchi Y, Ishikawa F. Anthocyanins from Purple Sweet Potato Ipomoea batatas Cultivar Ayamurasaki Suppress the Development of Atherosclerotic Lesions and Both Enhancements of Oxidative Stress and Soluble Vascular Cell Adhesion Molecule-1 in Apolipoprotein E-Deficient Mice. J Agr Food Chem. 2008;56(23):11485–92. 10.1021/jf801876n PubMed PMID: WOS:000261429000059. 18986148

[39] Liu Y, Li D, Zhang Y, Sun R, Xia M. Anthocyanin increases adiponectin secretion and protects against diabetes-related endothelial dysfunction. Am J Physiol Endocrinol Metab. 2014;306(8):E975–88. 10.1152/ajpendo.00699.2013 . 24595303

[40] Donato AJ, Morgan RG, Walker AE, Lesniewski LA. Cellular and molecular biology of aging endothelial cells. J Mol Cell Cardiol. 2015;89(Pt B):122–35. 10.1016/j.yjmcc.2015.01.021 ; PubMed Central PMCID: PMCPMC4522407. 25655936 PMC4522407

[41] Fratantonio D, Cimino F, Molonia MS, Ferrari D, Saija A, Virgili F, et al Cyanidin-3-O-glucoside ameliorates palmitate-induced insulin resistance by modulating IRS-1 phosphorylation and release of endothelial derived vasoactive factors. Biochim Biophys Acta. 2017;1862(3):351–7. 10.1016/j.bbalip.2016.12.008 . 28011403

[42] Song Y, Huang L, Yu J. Effects of blueberry anthocyanins on retinal oxidative stress and inflammation in diabetes through Nrf2/HO-1 signaling. J Neuroimmunol. 2016;301:1–6. 10.1016/j.jneuroim.2016.11.001 . 27847126

[43] Fratantonio D, Speciale A, Ferrari D, Cristani M, Saija A, Cimino F. Palmitate-induced endothelial dysfunction is attenuated by cyanidin-3-O-glucoside through modulation of Nrf2/Bach1 and NF-kappaB pathways. Toxicol Lett. 2015;239(3):152–60. 10.1016/j.toxlet.2015.09.020 . 26422990

[44] Speciale A, Anwar S, Canali R, Chirafisi J, Saija A, Virgili F, et al Cyanidin-3-O-glucoside counters the response to TNF-alpha of endothelial cells by activating Nrf2 pathway. Mol Nutr Food Res. 2013;57(11):1979–87. 10.1002/mnfr.201300102 . 23901008

[45] Han KH, Sekikawa M, Shimada K, Hashimoto M, Hashimoto N, Noda T, et al Anthocyanin-rich purple potato flake extract has antioxidant capacity and improves antioxidant potential in rats. Br J Nutr. 2006;96(6):1125–33. . 17181888 10.1017/bjn20061928

[46] Braunlich M, Slimestad R, Wangensteen H, Brede C, Malterud KE, Barsett H. Extracts, anthocyanins and procyanidins from Aronia melanocarpa as radical scavengers and enzyme inhibitors. Nutrients. 2013;5(3):663–78. 10.3390/nu5030663 ; PubMed Central PMCID: PMCPMC3705312. 23459328 PMC3705312

[47] Liobikas J, Skemiene K, Trumbeckaite S, Borutaite V. Anthocyanins in cardioprotection: A path through mitochondria. Pharmacol Res. 2016;113(Pt B):808–15. 10.1016/j.phrs.2016.03.036 . 27038533

[48] Xu JW, Ikeda K, Yamori Y. Inhibitory effect of polyphenol cyanidin on TNF-alpha-induced apoptosis through multiple signaling pathways in endothelial cells. Atherosclerosis. 2007;193(2):299–308. 10.1016/j.atherosclerosis.2006.09.006 . 17045269

[49] Liu W, Lu X, He G, Gao X, Xu M, Zhang J, et al Protective roles of Gadd45 and MDM2 in blueberry anthocyanins mediated DNA repair of fragmented and non-fragmented DNA damage in UV-irradiated HepG2 cells. Int J Mol Sci. 2013;14(11):21447–62. 10.3390/ijms141121447 ; PubMed Central PMCID: PMCPMC3856014. 24177565 PMC3856014

[50] Molnar J, Yu S, Mzhavia N, Pau C, Chereshnev I, Dansky HM. Diabetes induces endothelial dysfunction but does not increase neointimal formation in high-fat diet fed C57BL/6J mice. Circ Res. 2005;96(11):1178–84. 10.1161/01.RES.0000168634.74330.ed . 15879311

[51] Rajapakse AG, Yepuri G, Carvas JM, Stein S, Matter CM, Scerri I, et al Hyperactive S6K1 mediates oxidative stress and endothelial dysfunction in aging: inhibition by resveratrol. PLoS One. 2011;6(4):e19237 10.1371/journal.pone.0019237 ; PubMed Central PMCID: PMCPMC3081344. 21544240 PMC3081344

[52] Bhatt SR, Lokhandwala MF, Banday AA. Resveratrol prevents endothelial nitric oxide synthase uncoupling and attenuates development of hypertension in spontaneously hypertensive rats. Eur J Pharmacol. 2011;667(1–3):258–64. 10.1016/j.ejphar.2011.05.026 . 21640096

[53] Xia N, Daiber A, Habermeier A, Closs EI, Thum T, Spanier G, et al Resveratrol reverses endothelial nitric-oxide synthase uncoupling in apolipoprotein E knockout mice. J Pharmacol Exp Ther. 2010;335(1):149–54. 10.1124/jpet.110.168724 . 20610621

