# Supplementary material for: Boysenberry polyphenol inhibits endothelial dysfunction and improves vascular health
Source: PLoS One. 2018 Aug 14;13(8):e0202051. doi: 10.1371/journal.pone.0202051 (PMC6091942; doi:10.1371/journal.pone.0202051)
Supplement: S1 Fig — Wild-type mice were fed normal chow (NC) or a high fat diet (HFD). In some groups, boysenberry polyphenol (BP; 0.1% in the drinking water) was administrated in addition to the HFD (HFD+BP). A, B. Body weight (n = 6,8,8) and Food intake(n = 4,6,6)(A), systolic blood pressure, diastolic blood pressure, and heart rate(B) of indicated mice group(n = 6,6,7). C. Glucose tolerance test of indicated mice group(n = 6,6,7). D. Transcript for Cdkn1a as analyzed in aorta from indicated mice group(n = 5,5,5). E. Western blot analysis of total eNOS, and β-actin in the aorta. The right panel shows quantification of the total eNOS adjusted for β-actin(n = 13,13). Data were analyzed by the 2-tailed Student’s t-test (E), 2-way ANOVA followed by Tukey’s multiple comparison test (A, B, D), or Repeated measures followed by Tukey’s multiple comparison test(C). *P < 0.05; **P < 0.01. Values represent the mean ± SEM. (DOCX) [file pone.0202051.s001.docx]

**
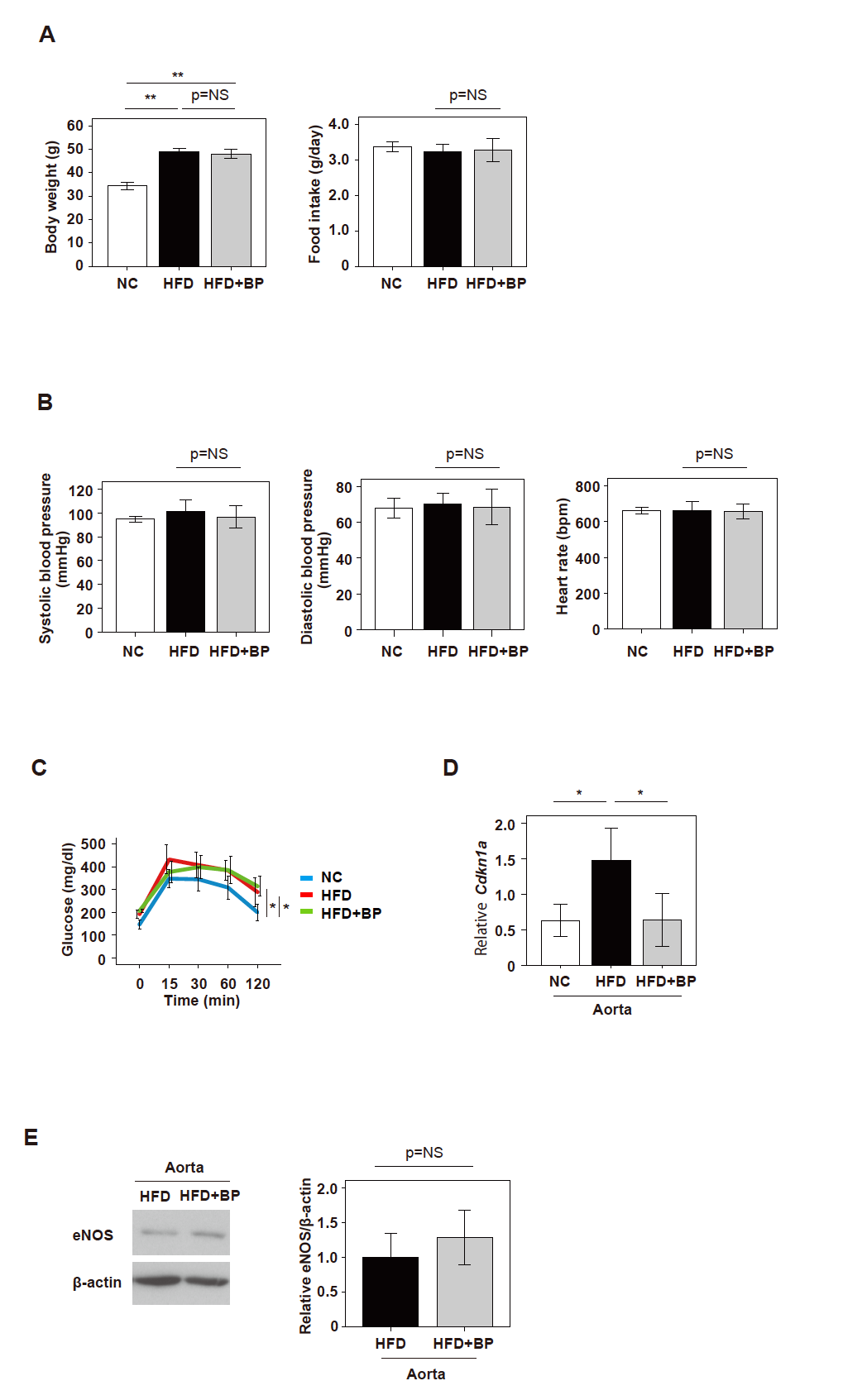
**

**S1 Fig Baseline characteristics of mice administrated with Boysenberry polyphenol**

Wild-type mice were fed normal chow (NC) or a high fat diet (HFD). In some groups, boysenberry polyphenol (BP; 0.1% in the drinking water) was administrated in addition to the HFD (HFD+BP). **A, B.** Body weight (n=6,8,8) and Food intake(n=4,6,6)(A), systolic blood pressure, diastolic blood pressure, and heart rate(B) of indicated mice group(n=6,6,7). **C.** Glucose tolerance test of indicated mice group(n=6,6,7). **D.** Transcript for *Cdkn1a* as analyzed in aorta from indicated mice group(n=5,5,5). **E.** Western blot analysis of total eNOS, and β-actin in the aorta. The right panel shows quantification of the total eNOS adjusted for β-actin(n=13,13). Data were analyzed by the 2-tailed Student’s t-test (E), 2-way ANOVA followed by Tukey’s multiple comparison test (A, B, D), or Repeated measures followed by Tukey’s multiple comparison test(C). *P < 0.05; **P < 0.01. Values represent the mean ± SEM.
