# Supplementary material for: Boysenberry polyphenol inhibits endothelial dysfunction and improves vascular health
Source: PLoS One. 2018 Aug 14;13(8):e0202051. doi: 10.1371/journal.pone.0202051 (PMC6091942; doi:10.1371/journal.pone.0202051)
Supplement: S2 Fig — Human umbilical vein endothelial cells (HUVECs) were treated with BSA (Con group), palmitic acid (200μM (for 1week culture) or 500μM (for 6hr culture)) (PA group), or PA (200μM (for 1week culture) or 500μM (for 6hr culture)) + BP (10 μg/ml) (PA+BP group). HUVECs were cultured with PA and/or BP for totally 1week for studies in Part A in S2 Fig and 6hrs for studies in Part B in S2 Fig. A. DAR-4M staining of HUVECs to detect nitric oxide (Scale bar = 100 μm). The right graph shows the relative fluorescence intensity (n = 5,5,5). B. Western blot analysis of phospho-eNOS(p-eNOS), eNOS and β-actin in HUVECs. The right panel shows quantification of the p-eNOS adjusted for eNOS (n = 4,4,4,4). Data were analyzed by 2-way ANOVA followed by Tukey’s multiple comparison test(A), or the 2-tailed Student’s t-test (B). *P < 0.05; **P < 0.01. Values represent the mean ± SEM. (DOCX) [file pone.0202051.s002.docx]

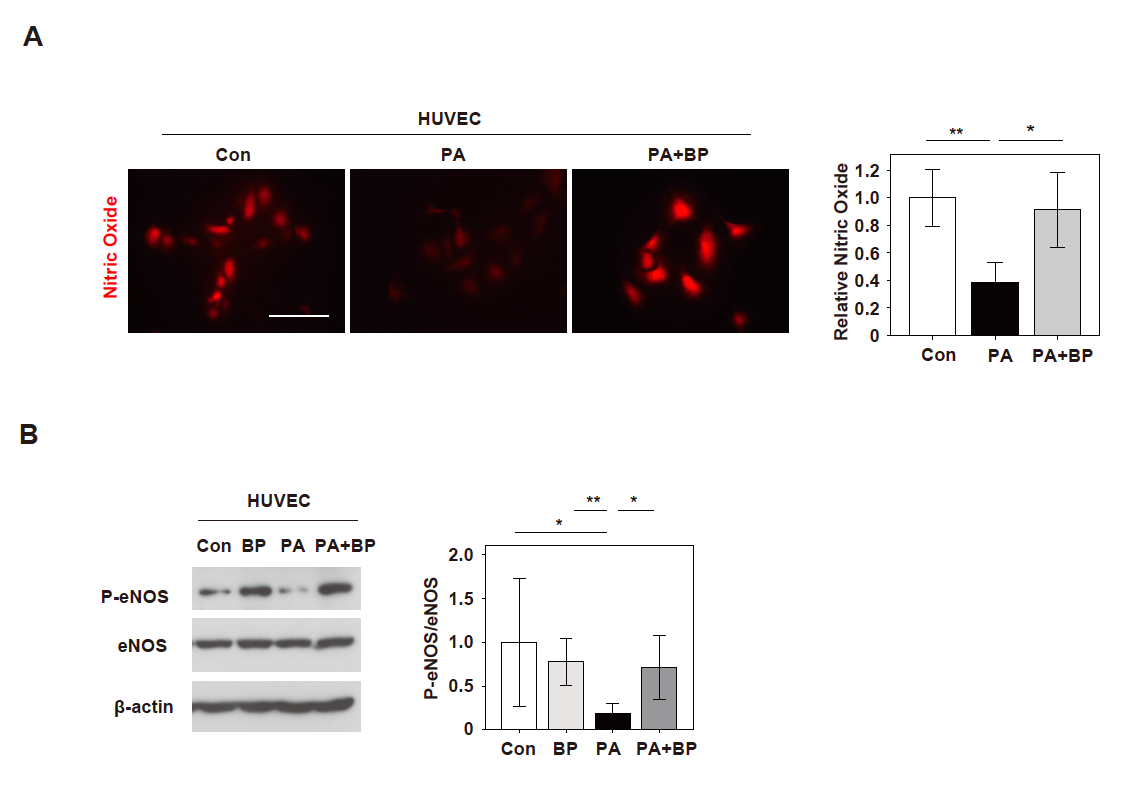


**S2 Fig Boysenberry polyphenol improves nitric oxide production in HUVECs also in chronic phase**

Human umbilical vein endothelial cells (HUVECs) were treated with BSA (Con group), palmitic acid (200μM (for 1week culture) or 500μM (for 6hr culture)) (PA group), or PA (200μM (for 1week culture) or 500μM (for 6hr culture)) + BP (10 μg/ml) (PA+BP group). HUVECs were cultured with PA and/or BP for totally 1week for studies in Supplementary Fig. 2A and 6hrs for studies in Supplementary Fig. 2B. **A.** DAR-4M staining of HUVECs to detect nitric oxide (Scale bar=100 μm). The right graph shows the relative fluorescence intensity (n=5,5,5). **B.** Western blot analysis of phospho-eNOS(p-eNOS), eNOS and β-actin in HUVECs. The right panel shows quantification of the p-eNOS adjusted for eNOS (n=4,4,4,4). Data were analyzed by 2-way ANOVA followed by Tukey’s multiple comparison test(A), or the 2-tailed Student’s t-test (B). *P < 0.05; **P < 0.01. Values represent the mean ± SEM.
