# Supplementary material for: Boysenberry polyphenol inhibits endothelial dysfunction and improves vascular health
Source: PLoS One. 2018 Aug 14;13(8):e0202051. doi: 10.1371/journal.pone.0202051 (PMC6091942; doi:10.1371/journal.pone.0202051)
Supplement: S3 Fig — A. High-performance liquid chromatography (HPLC) profile at 520 nm of anthocyanins (AC) from purified boysenberry polyphenol (BP). B. Classified anthocyanins (AC) were quantified using a cyanidin-3-glucoside standard. (DOCX) [file pone.0202051.s003.docx]

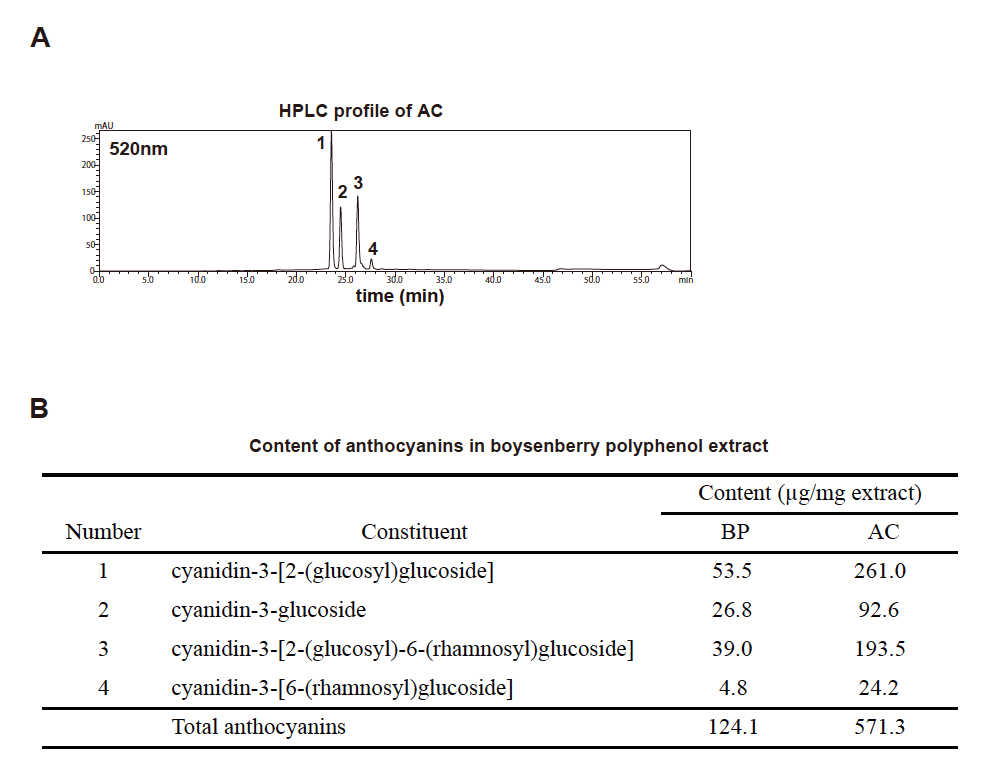


**S3 Fig Characterize of boysenberry polyphenols and anthocyanin fraction.**

**A.** High-performance liquid chromatography (HPLC) profile at 520 nm of anthocyanins (AC) from purified boysenberry polyphenol (BP). **B.** Classified anthocyanins (AC) were quantified using a cyanidin-3-glucoside standard.
